# Voracity, reaction to stings, and survival of domestic hens when feeding on the yellow scorpion (*Tityus serrulatus*)

**DOI:** 10.1590/1678-9199-JVATITD-2021-0050

**Published:** 2022-02-11

**Authors:** Gabriel Pimenta Murayama, Guilherme Ferreira Pagoti, José Paulo Leite Guadanucci, Rodrigo Hirata Willemart

**Affiliations:** 1 Institute of Biosciences, University of São Paulo (USP), São Paulo, SP, Brazil.; 2 School of Arts, Sciences and Humanities (EACH), University of São Paulo (USP), São Paulo, SP, Brazil.; 3 Institute of Biosciences, State University of São Paulo (UNESP), Rio Claro, SP, Brazil.

**Keywords:** Biological control, Buthidae, Natural enemy, Predator-prey interactions

## Abstract

**Background:**

Scorpionism is a worldwide problem that has already made thousands of victims, and multi-disciplinary approaches for controlling their populations are to be more successful. Hens are often mentioned as tools for controlling scorpions; however, systematic/experimental behavioral studies are not available. Moreover, there is no systematic information on the effect of scorpion venoms on hens. Using the venomous yellow scorpion *Tityus serrulatus*, the present study aimed to clarify the following aspects: (1) voracity of hens, (2) how hens react when stung, (3) the effect of scorpion stings on hen behavior during attacks, and (4) hen survivorship after feeding on scorpions.

**Methods:**

We attracted hens with corn powder, offered them scorpions and then recorded the hen-scorpion interaction. To test the effects of the sting we manually removed the scorpion’s telson.

**Results:**

We found that some hens ate up to six scorpions within minutes. By means of an ethogram and drawings, we showed that they exhibited several aversive behaviors when capturing scorpions. Removal of the scorpion telson stopped the aversive reactions, which was not observed in the control group. Finally, hens did not exhibit atypical behaviors after 1, 7 and 30 days and were all alive after 30 days.

**Conclusion:**

This is the first empirical and video recorded study providing evidence that hens are clearly affected by scorpion venom but do not die. Therefore, they may have potential to be used in biological control of these arthropods.

## Background

Scorpionism is a widespread problem around the world, especially in regions of Africa, India, Mexico, Australia and South America [1,2]. In Brazil, the species responsible for the majority of accidents and deaths is the yellow scorpion *Tityus serrulatus* [3,4]. This species is originally from the Southeast of the country, but its distribution has expanded [5,6,7,8]. These scorpions are currently found in many States [9,] and even in other countries such as Argentina and Bolivia [6,10,11]. Furthermore, *T. serrulatus* can reproduce by parthenogenesis [12] and fast for long periods [13], making it an even more complex pest to control. Unfortunately, very few studies have addressed scorpion control [14,15,16].

There are many methods for controlling pests, such as using plant extracts [17], fungi, bacteria [18], pesticides [19] and biological control by animals [20]. In some cases, biological control is a good alternative because it can be a cheap and nontoxic method to the environment [21,22]. For a successful and efficient biological control, the voracity of the introduced predator is an essential trait to be considered [23], obviously considering prey abundance. For example, an egret can consume around 100-150 grams of fly larvae each day in waste dump’s areas [24], and a single ladybird beetle can eat around 100 aphids in 24 hours [25,26]. The more abundant the prey, the more voracious the predators used in pest control must be.

Toxic and venomous prey have to be dealt with care by the predator since prey can either kill or negatively affect predators’ behavior [27,28]. However, some predators are able to remove or avoid toxic body parts of prey [29,30] and others are immune to the prey venom [31]. Thus, predators used to control prey must have one of such characteristics, ultimately surviving attacks. 

In scorpions, the possibility of using predators to help controlling their population locally has never been systematically tested. There are many animals that feed on scorpions and Polis et al. [32] have provided an extraordinary compilation of scorpion predators. The list includes other arachnids [33,34], mantids, chilopods, frogs, lizards, bats, birds and others. Birds are predators of arthropods in general [35,36,37,38], including scorpions [39,40].

As many birds, the hen *Gallus gallus domesticus* is an omnivore animal [41], easily found around the world and cheap to obtain and rear. It easily adapts to synanthropic environments where most accidents with yellow scorpions occur. If the requirements mentioned above are met, hens could have potential to at least help controlling local populations of yellow scorpions. It is a common but controversial saying that hens are good to control scorpions. Based on a single person interviewed and from eight scorpions offered to a single hen that fed on the scorpions, Dias et al. [42] concluded that hens are scorpion predators and doubtless excellent for controlling scorpion populations. Cruz et al. [43] reported that the mayor of Aparecida city (in São Paulo State, Brazil) distributed hens to the population as an attempt to control scorpion infestation, but the efficacy was never tested. The Brazilian Ministry of Health [44] reported that hens are not efficient in controlling scorpions, but do not cite studies. There is definitely a need for a more detailed study on the predatory interaction between hens and scorpions. Here we studied the interaction between the hen *G. gallus domesticus* and the yellow scorpion *T. serrulatus* aiming to understand: (1) voracity of hens, (2) how hens react when stung, (3) the effect of the sting on hen behavior during attacks, and (4) hen survivorship after feeding on scorpions. 

## Methods

### General procedures

We collected adult scorpions in the city of Santa Gertrudes (State of São Paulo, Brazil) between January and April 2019. We maintained scorpions in terraria (45 length x 21 width x 30 height cm) with water *ad libitum* and fed them cockroaches (*Periplaneta americana*) every 15 days. We carried out all the experiments in a private farm with hens in the city of Paulínia (State of São Paulo, Brazil) in May 2019, except the one described in the section “Hen response while feeding on scorpion with and without telson”. The hens (*Gallus gallus domesticus*; n ​​= 50) were maintained in an isolated area of 266 meter². Hen response while feeding on scorpion with and without telson meters^2^ with low trees, shelter and substrate covered by soil, grass, tree trunks and pieces of wood. For experiment in the section “Hen response while feeding on scorpion with and without telson”, we collected scorpions in the city of Botucatu (State of São Paulo, Brazil) in September 2020. We carried out this experiment in another private farm with hens in the city of Paulínia (State of São Paulo, Brazil) in October and November 2020. The hens used in this experiment were maintained as described above, but in this farm the hens had an area of 550 meters^2^.

We used hens that were being fed daily with enriched bird food and corn kernels. Hens had never been seen interacting with scorpions before the experiment and had never been seen in the surroundings of the experimental areas. Before starting the experiments, we observed the hens for about 30 minutes. During this period, we observed the hens walking, approaching us, scratching themselves and eating normally. We observed hen-scorpion interactions between 1 and 4 PM for two consecutive days. We made the observations where the hens were being maintained to minimize stress due to translocation. Because the hens had been reared free and in group, we also did not individualize them in cages to avoid stress that could lead to unusual behaviors.

### Reaction to scorpion stings

To describe the interactions between hens and scorpions, we attracted hens with corn powder. When at least one hen was approximately 2 m from us, we released, using tweezers, a live scorpion at a distance of approximately 30 cm from the hen’s body. An average of 3.4 hens (minimum 1, maximum 10 hens) were close to us when the first hen captured the scorpion. We offered a total of 61 scorpions. We recorded (SonyHandycamHDR-XR55) all hen behaviors, from the moment we offered the scorpion until it was completely swallowed by one of the hens. We scored whether the hen was stung or not (see “Video analyses” section), hen reaction to scorpion stings (in behavioral categories) and the number of hens that fed on scorpions.

Video analyses

By analyzing the videos, we verified which hens had been stung. Unfortunately, due to the quality and distance at which the records were made, we were unable to detect the sting penetrating in the hen integument. We inferred that hens were stung because: (1) the specific hen behaviors we have observed occurred right after the hen made contact with the scorpion or swallowed it and (2) hens in their regular activities before the experiments had never behaved these ways. Therefore, these behaviors were used as proxies of a scorpion sting throughout the study and this text. The possibility that some hens were stung but did not show any detectable reaction cannot be ruled out. We then analyzed the videos and built an ethogram of the predatory interaction. 

Hen survival after feeding on scorpions

To investigate the venom´s effect on hens, we monitored hens´ behavior one, seven and 30 days after feeding on the scorpions. We scored whether the hens behaved as previously to the exposure to scorpions, presented atypical behaviors or died.

### Hen response while feeding on scorpionS with and without telson

To test if hen’s behaviors were actually in response to the scorpion sting, we removed the telson by cutting the constriction between the telson and the metasoma with scissors. We then offered 26 scorpions without telson and 40 control scorpions (with telson) to the hens 10 min after the cut. Because of the limited number of hens, we used a repeated measures design in which the same hens were exposed to both treatments. Therefore, because we knew from previous observations (see “Reaction to scorpion stings”) that hens would probably react aversively in the control group, here we first offered the treatment group to minimize possible effects of previous aversive reactions. The methods to attract, offer the scorpion and record the interaction between the hens and scorpions were the same as in “Reaction to scorpion stings”. However, in this case the area used for observations had 25 meters^2^. We divided the number of scorpions to which a hen had at least one aversive behavior by the number of scorpions it attacked. We compared only hens that captured prey in both treatments. We ran a Wilcoxon test comparing the treatment vs the control group. Because a single hen sometimes interacted with several scorpions, we also compared the number of scorpions that hens attempted to prey upon between the control and treatment groups to control for the probability of hens having aversive behaviors.

## Results

### Description of scorpion capture

In 41 out of 61 interactions we were able to identify which hen ingested the scorpion. In the remaining 20 interactions, we only witnessed hens holding dead scorpions but failed to register ingestion. It is, however, most likely that these scorpions were also ingested. When the scorpion is alive it tries to hold the hen with pedipalps, we then considered that the scorpion was dead when we saw the scorpion swing on hens’ beak. While handling the scorpion, the most common behavior observed was to hold it within the beak and hit it against the substrate before ingesting. Hens consumed between 1 and 3 (n = 15), 6 (n = 2) and 7 (n = 1) scorpions. 

### Reaction to scorpion stings

**Figure 1.**
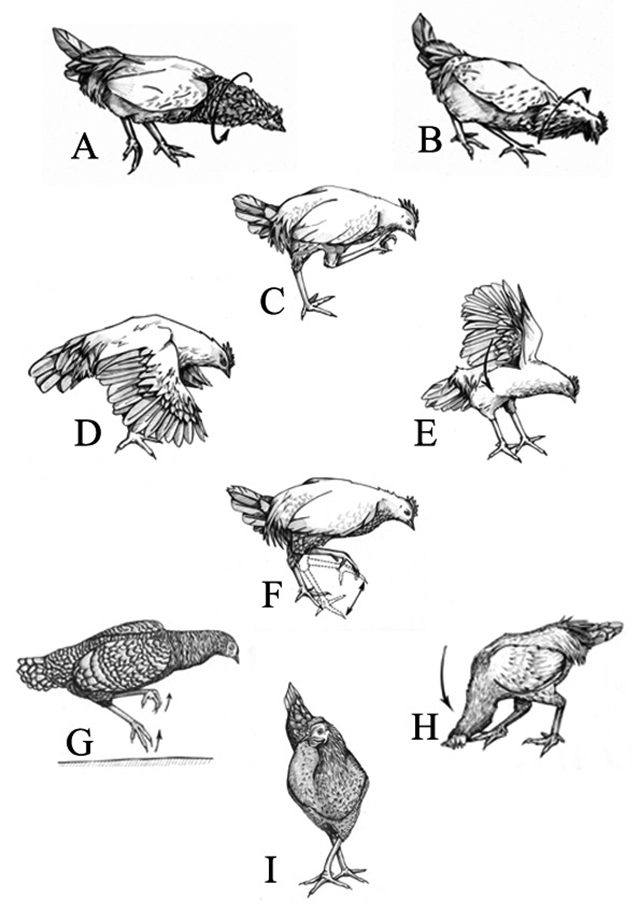
Representation of the aversive behaviors of the hen *Gallus gallus domesticus* when interacting with the yellow scorpion *Tityus serrulatus*. (**A**, **B**) shaking head; (**C**) scratch the beak/face with feet; (**D**, **E**) open wings; (**F**) stomp; (**G**, **I**) - jump; (**H**) - scratch the beak/face on substrate.

Hens were stung in 29 occasions out of 61 scorpions offered (nineteen interactions, some hens were stung more than once). Most hens (n = 27) that were stung by scorpions immediately released them. In addition, they presented certain typical post-sting behaviors, such as shaking their heads (Fig. 1A and 1B) and scratching their beaks and/or their faces with their feet (Fig. 1C, see other behaviors and descriptions in Table 1 and in the video of the Additional file 1). Most hens performed more than one of these behaviors when capturing a scorpion that stung. The other 42 did not exhibit any of these listed behaviors when attempting to capture the offered scorpion. None of these behaviors had beenn observed before the experiments.

**Table 1.** Aversive behaviors of the domestic hen *Gallus gallus domesticus* after being bitten by the yellow scorpion *Tityus serrulatus*.

Category	Description	N
Shaking head	Move the head quickly and successively latero-laterally (Fig. 1, A and B)	29
Scratch the beak/face with feet	Rub the beak and/or face with one foot at least once (Fig. 1 C)	14
Open wings	Extend one or two wings to the side, making dorso-ventral movements with one or two wings (Fig. 1, D and E)	8
Stomp	Raising and lowering successively one foot and then the other in repeated movements (Fig. 1 F)	2
Jump	Take both feet off of the substrate, sometimes crossing the legs and leaning the body to one side (Fig. 1, G and I)	2
Scratch the beak/face on substrate	Rub the beak laterally against the substrate (Fig. 1 H)	2

### Hen survival after feeding on scorpions

Among hens that displayed one of the above-mentioned aversive behaviors, hens were stung on average 1.7 times (std dev = 0.9, min = 1; max = 4) during the descriptive part of the study. All of these hens (n = 18) were alive, feeding and showing no atypical behavior one, seven and 30 days after feeding on scorpions. During the experiment below (with vs. without telson), a single hen was stung up to six times in 60 minutes and was still alive after the experiment with all the hens finished. We did not monitor it afterwards.

### Hen response while feeding on scorpionS with and without telson

Hens in the treatment group attacked 4 scorpions (median, min = 1, max = 12) and those in the control group attacked 2.5 scorpions (median, min = 1, max = 9) (Wilcoxon test; W = 14; P = 0.426). Hens showed aversive reactions in about half their interactions with scorpions with telson (median = 0.55; min = 0; max = 1) but none had aversive reactions with scorpions without telson (Wilcoxon test; W = 36; P = 0.008).

Being stung did not prevent some hens to keep attempting to capture scorpions. All six hens (during this experiment) that released the scorpion after being stung tried to recapture right after having dropped it. Being stung also did not prevent them from attacking scorpions: the most striking examples are three individuals that attacked 6, 9 and 11 scorpions after being stung in less than 40 min.

## Discussion

We have shown that hens are voracious scorpion predators and that they may react aversively in different ways to scorpion defenses. Since hens have nociceptors (pain receptors) in the beak [45,46], these aversive behaviors are probably due to the pain caused by the *T. serrulatus* sting. We have also provided experimental evidence that it is probably the sting that causes hens reactions and have shown that hens do not exhibit atypical behaviors after 1, 7 and 30 days and are all alive after 30 days. 

One important criterium a predator has to meet to be a potential good species to control a pest is being voracious towards the specific prey. Hens clearly fit this criterium, having attacked up to 11 scorpions in less than 40 min. Were the hens not grouped with other hens, they would probably eat larger quantities. Other evidences of voracity are that hens would quickly move towards the given scorpions and would often try to steal from their counterparts. According to the author's observations, hens clearly preferred scorpions over of powder corn.

A second criterium is to somehow not let stinging by the prey hamper the attack. Forty-two out of 61 scorpions did not cause any aversive reaction, suggesting they did not get to sting. This suggests hens are efficient scorpion predators. When pressed against the substrate or manipulated, scorpions defend themselves mainly by driving their pedipalps and metasoma towards the aggressor, pinching and stinging [47,48]. Contrary to what has been reported by Dehghani et al. [49] in interactions between Iranian species of scorpions and hens, the yellow scorpion often successfully stung the hens, which reacted in different ways (Table 1; Fig. 1A-1I). However, being stung did not prevent hens to continue the attack and chase other scorpions afterwards. As has been previously reported, reacting aversively when stung by scorpions does not mean aborting the attack ([50] example with lizards). All hens survived the attacks despite being stung several times in less than an hour. The venom of *T. serrulatus* is a cocktail that includes powerful neurotoxins [51,52]. Carcamo-Noriega et al. [53] found that a dose of 125 μg/kg and the injection of 100 μL of the scorpion *Centruroides sculpturatus* isolated venom were lethal to hens. The controlled quantity of venom used, the fact that Carcamo-Noriega et al. [53] used isolated toxins and the different scorpion species might explain the distinct results. Scorpions can control the amount of venom used [54] and because in our experiment it was a life-or-death situation, it is reasonable to assume that scorpions did not use dry stings [54]. 

Finally, a third criterium is that predators have to survive most of the attacks towards venomous prey. Since all the hens were alive and sound 30 days after the experiment, these birds meet the requirement of not getting killed by the yellow scorpion. Hens are not the only predator that seems to be immune to stings of the yellow scorpion. Jared et al. [55] have shown that the toad *Rhinella icterica* also survive after feeding on *T. serrulatus*, with the advantage of being a nocturnal animal and therefore with a great overlap between the activity periods of toads and scorpions, but the disadvantage of being harder to obtain and maintain in synantropic environments*.*

There are several requirements for a predator to be an adequate species to control specific prey. We have shown that the most basic ones have been met by *Gallus gallus domesticus*, namely being a voracious predator of the yellow scorpion and being immune to the venom even if hens often react aversively to the sting. It is common sense that hens are good for biological control of scorpions. However, hens are said to be diurnal and yellow scorpions are mainly nocturnal [56]. Interestingly, hens may also have nocturnal activity. We have monitored 17 individuals between 18h-6h for two days and have data showing that, at night, they have different behaviors such as cleaning, scratching and walking. While walking, they sometimes wake up other hens. We also have data, that will also be published elsewhere, showing that hens will also feed on scorpions at night (Murayama, Pagoti and Willemart, unpublished data). Therefore, we have shown that at least the most fundamental requirements for a species to be used to control a specific prey have been met by hens.

## Conclusion

We have shown that hens meet important criteria if they are to be used to control local populations of scorpions. They may consume several individuals and although the sting causes hens to react aversively, they all survive and behave normally afterwards. 

## Supplementary material

The following online material is available for this article:

Additional file 1. Video showing aversive and non-aversive behaviors of hens.
