# Supplementary material for: Voracity, reaction to stings, and survival of domestic hens when feeding on the yellow scorpion (Tityus serrulatus)
Source: J Venom Anim Toxins Incl Trop Dis. 2022 Feb 11;28:e20210050. doi: 10.1590/1678-9199-JVATITD-2021-0050 (PMC8833830; doi:10.1590/1678-9199-JVATITD-2021-0050)
Supplement: Additional file 1. [file 1678-9199-jvatitd-28-e20210050-s1.zip › 1678-9199-jvatitd-28-e20210050-s1.pdf]

J Venom Anim Toxins incl Trop Dis

https://doi.org/10.1590/1678-9199-JVATITD-2021-0050

Supplementary Material to "Voracity, reaction to stings, and survival of domestic hens when feeding on the yellow scorpion (*Tityus serrulatus*)"

Additional file 1. Video showing aversive and non-aversive behaviors of hens.
